# The effect of medical students’ gender, ethnicity and attitude towards poetry-reading on the evaluation of a required, clinically-integrated poetry- based educational intervention

**DOI:** 10.1186/1472-6920-14-188

**Published:** 2014-09-15

**Authors:** Mordechai Muszkat, Orly Barak, Gadi Lalazar, Bracha Mazal, Ronen Schneider, Irit Mor-Yosef Levi, Matan J Cohen, Laura Canetti, Arie Ben Yehuda, Yaakov Naparstek

**Affiliations:** The Department of Medicine, Hadassah-Hebrew University Medical Center, Jerusalem, 91120 Israel; The Department of Psychology, Hebrew University, Jerusalem, Israel

**Keywords:** Art-based intervention, Poetry, Medical humanities, Medical education

## Abstract

**Background:**

Art -based interventions are widely used in medical education. However, data on the potential effects of art-based interventions on medical students have been limited to small qualitative studies on students’ evaluation of elective programs, and thus their findings may be difficult to generalize. The goal of this study is to examine, in an unselected students’ population, the effect of students’ gender, ethnicity and attitude towards poetry on their evaluation of a clinically-integrated poetry-based educational intervention.

**Methods:**

A required Clinically- Oriented Poetry-reading Experience (COPE) is integrated into the 4^th^ year internal medicine clerkship. We constructed a questionnaire regarding the program’s effects on students. Students completed the questionnaire at the end of the clerkship. We performed a Confirmatory Factor Analysis, and examined the relationship between students’ evaluation of the program and students’ ethnicity, gender, attitude towards poetry-reading, and the timing of the program (early/late) during the fourth year.

**Results:**

144 students participated in the program, of which 112 completed the questionnaires. We identified two effect factors: “student-patient” and “self and colleagues”. The average score for “student-patient” factor was significantly higher as compared to the “self and colleagues” factor.

Evaluation the “student- patient” effect factor was higher among Arab and Druze as compared to Jewish students. Students’ attitude towards poetry-reading did not correlate with the “student-patient” effect, but correlated with the “self and colleagues” effect. The evaluation of the “self and colleagues” effect was higher among students who participated in the program during their second as compared with the first clerkship. Students’ gender was not associated with any of the effects identified. Students favored obligatory participation in COPE as compared with elective course format.

**Conclusions:**

According to students’ evaluation, a format of integrated, obligatory poetry-based intervention may be suitable for enhancing “student-patient” aims in heterogeneous student populations. The higher evaluation of the “patient-student” effect among Arab and Druze as compared to Jewish students may be related to cultural differences in the perception of this component of medical professionalism. Further research can provide insight into the effect of cultural and ethnic differences on actual empathy of medical students in patient encounters.

## Background

Art-based interventions have been used for various aims in undergraduate medical education. A major aim of art-based interventions in medical education is to enhance medical students’ empathic skills and awareness of patients’ experiences [1–3]. This is based on the assumption that literature and poetry regarding illness experiences can expose medical students to patients’ narratives, thus helping students gain a greater awareness of the patients’ perspectives [1]. In addition, art-based interventions have been suggested to enhance medical students’ self-reflection, communication and collaboration with colleagues, and understanding of physician-society relationships [2]. However, while programs in medical humanities and in narrative medicine have existed in many medical schools [3], evaluation of art-based interventions for undergraduate medical students has been limited [4], and includes mainly qualitative evaluation of elective medical humanities courses [4]. These studies provide important insights into students’ perceptions of the effects of art-based interventions. Such effects include, in addition to increased awareness of patients’ experiences of illness, enhanced capabilities of communication, analysis, presentation, writing and clinical reasoning, imagination, observation, and awareness of language [5–9]. The insights provided by qualitative studies of elective courses could serve larger quantitative studies in unselected students’ populations. However, no study has thus-far reported on the evaluation of a clinically-integrated art-based intervention in an unselected students’ population. Consequently, the effect of factors that can potentially affect curriculum planning, such as diversity in students’ ethnicity, gender, and attitude towards the arts, as well as the timing of the program during medical studies, have not been adequately evaluated.

### Study purpose

The aim of this study is to determine whether the evaluation of the effects of an integrated, obligatory poetry-based intervention in a heterogenic student population is related to diversity in students’ characteristics such as ethnicity, gender and attitude towards poetry-reading as well as to the timing of the program during medical studies.

## Methods

### Design

The study design employed cross sectional study including an anonymous survey. The investigators distributed copies of the questionnaires to students and students collected the questionnaires and delivered them to the investigators in closed envelopes.

The study was approved by the ethics committee for The Use of Human Subjects in Research for Medicine, Dental Medicine and Science Faculties, Hebrew University of Jerusalem.

### Population

The study included all 4^th^ year medical students who attended the internal medicine clerkship in all three medical wards at a single teaching hospital during three consecutive academic years (2008, 2009 and 2010).

### Setting

The medical school program is a 6-year program. Students’ first extensive clinical studies occur during the fourth year of medical school which includes 2 clinical clerkships: A 12-week clerkship in internal medicine and an 8 week clerkship in pediatrics. The clerkships in medicine and pediatrics are conducted in small groups of 8-10 students. One half of each class begins with the internal medicine clerkship, and continues to pediatrics and half of the class starts in pediatrics and proceeds to a clerkship in internal medicine. For each student’s group the order of clerkships and allocation of teaching hospitals is determined randomly at the beginning of the fourth year.

A poetry-based educational intervention, [Clinically- Oriented Poetry- reading Experience (COPE)] is included in the clerkship in the three medical departments at a single teaching hospital. These departments teach an average of 48 students during each academic year (48, 46, 50 students in 2008, 2009 and 2010, respectively).

COPE includes five bi-weekly meetings and was conducted according to the methodology described in our preliminary report [10], with modifications that included extending meeting length from 1 hour to 1.5 hours.

### COPE development

We conducted a pilot of the program in a single teaching hospital over the course of two academic years (2008 and 2009). During the first year of the pilot (2008) the content and structure of the program were established, and the evaluation questionnaire was constructed. 48 students participated in the program during 2008, of which 45 filled the questionnaires. Data from 44 of these students were available for an initial report on the activity, its aims, structure and content [10]. This report did not include factor analysis nor the analysis of the effect of students’ characteristics on the evaluation of the program.

### COPE facilitators

COPE’s facilitators (M.M., O.B., G.L., B.M., R.S., I.M.L.) are physicians, board certified in internal medicine, whom students meet during the clerkship at ward rounds and patients’ consultations prior to the beginning of COPE.

The initiator of the program (M.M.) participated in the basic and advanced training in Narrative Medicine (Columbia University workshops). Facilitators’ training included observation at other facilitators, preparatory discussions before and reflective meetings after observations.

Each student group worked with the same facilitator during all sessions, to enable the facilitator and students to establish trust and continuity in their relationship. In order to minimize variability among facilitators, facilitators met prior to each session for detailed discussion of the poems, and to plan the outline of the activity and especially expected questions.

### Poems used in COPE

Five poems were used in the program. Poems were chosen by the program’s initiator and facilitators. In each session a different poem was discussed. The order of poems was the same for all groups. All poems were in Hebrew.

Poems used reflected experiences such as illness, loss and death, patient-caregivers’ encounters, as well as experiences related to cultural and interethnic heterogeneity among patients and caregivers.

At the beginning of each meeting a poem was read and discussed, including the poem’s story line and language. Students were then asked to describe (usually in writing, for approximately 10 minutes) an experience they had had, or an event they had witnessed, that they found to be relevant to the experience recounted in the poem. Students’ experiences were often related to their encounters with patients and families, often reflecting ethnically and culturally heterogeneous Israeli patient population. During the discussions, students had an opportunity to share their experiences and to respond to other students in the group. Towards the end of the meeting the facilitator would summarize the discussion, often linking the students’ narratives to the poem.

### Instrument development

The study questionnaire was developed and iteratively revised over several months during the pilot period [11, 12]. A list of the program’s potential impacts was prepared, based on qualitative studies evaluating art-based interventions [5–9], as well as on the medical literature regarding the desired and potential effects of literature and medicine and narrative medicine programs [1–3]. COPE’s facilitators and an educational psychologist (A.L.) were consulted in clarifying the language of the questionnaires. Ultimately, 9 items were chosen that reflected COPE’s desired effects. Two of these items were negatively worded. Three additional items were added to address: the preferred mode of students’ participation (as an elective/required course), and students’ individual attitudes towards poetry-reading (Table 1). Each statement was graded on a Likert scale between 1 (“strongly disagree”) and 7 (“strongly agree”). In addition, the questionnaires included data on students’ age, gender and ethnicity.

**Table 1:** **Students’ evaluation of the program (years 2009-2010, n = 67) [Average ± SD, scale of: 1 (strongly disagree) to 7 (strongly agree)]**

***Item***	***Impact of the program: The program…***	Average ± SD
1	Increased my awareness of how patients feel	4.52 ± 1.90
2	Did not affect my awareness of patients’ emotions	3.06 ± 2.08
3	Increased my understanding of “what it is like to be a patient”	4.94 ± 1.81
4	Helped me to better understand the interpersonal components of my role as a physician	4.78 ± 1.70
5	Motivated me to increase my efforts towards patients’ communication	3.47 ± 1.80
6	Provoked conversations among students in topics that are related to the clinical experiences	4.48 ± 1.91
7	Did not affect students’ communication regarding clinical experiences	3.48 ± 2.02
8	Increased a sense of partnership among students	4.25 ± 1.79
9	Helped me process the emotional impact of my experiences in the medical ward	4.21 ± 1.70
	Participation should be obligatory	4.34 ± 1.79
	The program should be offered to students as an elective course*	3.05 ± 1.84
	Generally, I enjoy reading poetry	4.52 ± 2.06

*P < 0.001, as compared to “participation should be obligatory”.

### Data collection

Students completed the questionnaire at the end of the internal medicine clerkship. The questionnaires were anonymous, and physicians involved in the program were not present in the room while the questionnaires were distributed to students.

### Data analysis

#### Socio-demographic variables, attitude towards poetry and program’s timing

Gender and ethnicity were defined according to self-reporting. Students’ ethnicity was defined as “Israeli Jews” or as “Arabs and Druze”. Attitude towards poetry was defined according to student’s individual responses to the statement: “Generally, I enjoy reading poetry” (above the median or median and below). The timing of the intervention during the fourth year was defined as “early” if students’ had COPE during their first 4^th^ year clerkship, or “late” if students had COPE during their second 4^th^ year clerkship.

### Statistical analysis

Students’ responses to the questionnaires are presented as mean ± SD. Student’s t test was used to evaluate the differences in scores according to gender, ethnicity, timing during studies, and according to the attitude towards poetry (students with higher than the median as compared to median and lower score on: “Generally, I enjoy reading poetry”). Chi square test was used to examine the distributions of gender and ethnicity among students who completed the questionnaires and students who did not complete the questionnaires.

Confirmatory Factor Analysis was conducted on the full dataset. Cronbach alpha was used to characterize the internal reliability of each of the factors that emerged from the factor analysis and to assess the extent to which each item contributed to the overall reliability of the factors [11, 12]. According to the content of the items two factors were proposed: Factor 1 including items 1, 2, 3, 4 and 5, and factor 2 including items 6, 7, 8 and 9. Based on their content these factors were labeled “student-patient” and “self and colleagues”, respectively.

The unadjusted factor scores were compared according to students’ gender, ethnicity, attitude towards poetry, and the timing of intervention using the Student’s t test. The paired Student t-test was used to examine the differences between the two factors and between different items among students. The correlations between items regarding mode of participation and the scores of factor 1 and 2 were estimated by calculating the Pearson correlation coefficients.

A p <0.05 was considered significant. All statistical calculations were performed using SPSS 21.0 for Windows, and for CFA analyses we used Amos 21.0 for Windows.

## Results

### Subjects

One hundred and forty four 4^th^ -year medical students participated in the program between 2008-2010, 78 (54.2%) were men and 66 (45.8%) women, 123 Jewish (85.4%), 17 Arab and 4 Druze students (14.6%).

One hundred and twelve students (77.8%) completed the questionnaires. The mean age of students who completed the questionnaires was 26.38 ± 3.2 years (mean ± SD), 57 (57.6%) men and 42 (42.4%) women. 13 students did not provide gender data. Ethnicity of students who completed the questionnaires was: 71 (82.6%) Jews, 13 Arabs and 2 Druze (17.4%). 26 students did not provide data regarding ethnicity. There was no significant difference in the distributions of gender and ethnicity between students who completed the questionnaires and students who did not complete the questionnaires (p = 0.694, p = 0.852, respectively, Chi square test). 45 of the students participated in the study during 2008 (of which 44 contributed data to a preliminary report [10]), and 22 and 45 students participated in the study during 2009 and 2010, respectively. Individual items scores of the 67 students who participated in the program during 2009-2010 are presented in Table 1.

### Confirmatory factor analysis

According to our hypothesis and to the content of the items two factors were proposed: Factor 1 including items 1, 2, 3, 4 and 5, and factor 2 including items 6, 7, 8 and 9. Based on their content these factors were labeled “student-patient” and “self and colleagues”, respectively.

In reporting the results of CFA we followed the guidelines suggested by Raykov et al. [13] and by Hu and Bentler [14].

For the 67 students who participated in the study during 2009 and 2010 (not including the pilot at year 2008) the internal consistency for all items in the questionnaire was very good: α = 0.91. For factor 1 the internal consistency was very good: α = 0.91. For factor 2 the internal consistency was good: α = 0.80.

A Confirmatory Factor Analysis including these 67 subjects showed the model fits: the χ2/df = 2.001 which is under the upper limit of 5, NFI = 0.869 shows almost good fit, TLI = 0.872, also almost a good fit, the CFI = 0.926 good fit and RMSEA = 0.123 just above the upper limit of 0.10.

In order to further evaluate the questionnaire, CFA was performed for all students who participated in the program (2008-2010, n = 112). Similarly to the results of the CFA in 67 students , CFA in the entire 112 students who participated in the program 2008-2010, for all items in the questionnaire, the internal consistency was very good: α = 0.91*.* For Factor 1 the internal consistency was very good: α = 0.90. For factor 2, the internal consistency was good: α = 0.84.

A Confirmatory Factor Analysis (n = 112) showed that the model was good: the χ2/df = 2.202 which is under the upper limit of 5, NFI = 0.911 shows a good fit, TLI = 0.910, also a good fit, the CFI = 0.948 almost very good fit and RMSEA = 0.104 just above the upper limit of 0.10. Standardized estimates in Confirmatory Factor Analysis of the questionnaire are presented in Table 2. The average score on factor 1 in the 112 participants was 4.87 ± 1.48 and the score on factor 2 was 4.50 ± 1.56. Factor 1 was significantly higher as compared with factor 2 (p = 0.002).

**Table 2:** **Standardized estimates in confirmatory factor analysis of the questionnaires (2008-2010, n = 112)**

	Item	Factor 1	Factor 2
	***The program…***	(Student-patient)	(Self and colleagues)
1	Increased my awareness of how patients feel	0.90	
2	Did not affect my awareness of patients’ emotions (positive)	0.719	
3	Increased my understanding of “what it is like to be a patient”	0.765	
4	Helped me to better understand the interpersonal components of my role as a physician	0.866	
5	Motivated me to increase my efforts towards patients’ communication	0.770	
6	Provoked conversations among students in topics that are related to the clinical experiences		0.867
7	Did not affect students’ communication regarding clinical experiences (positive)		0.825
8	Increased a sense of partnership among students		0.648
9	Helped me process the emotional impact of my experiences in the medical ward		0.709
	**Variance explained by factor (%)**	35.03%	32.07%

### Factors associated with students’ evaluation of the program’s effects

Student-related factors associated with the factor scores were studied in all the 112 students who participated in the program.

### Students’ gender and the evaluation of the program’s effects

**Figure 1:**
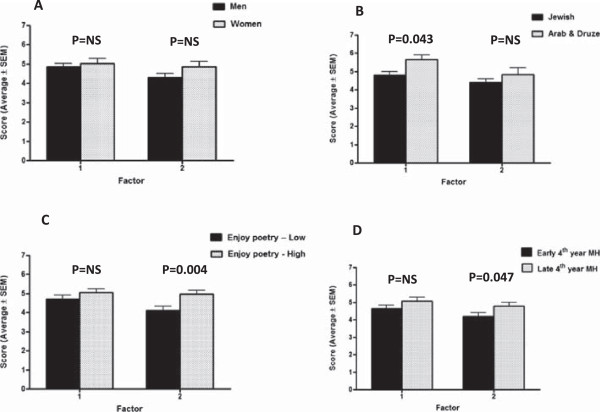
**Unadjusted scores (mean ± SEM) of factor 1 (“student-patient”) and factor 2 (“self and colleagues”) according to students’ characteristics (p values, Student’s-t test).** Panel 1**A**: Differences between men and women, Panel 1**B**: Differences between Arab and Druze vs Jewish students, Panel 1**C**: Differences between students with a score >5, as compared to students with a score ≤5 for: “Generally, I enjoy reading poetry”. Panel 1**D**: Differences between students who participated in the program during their first and second 4^th^ year clerkship.

There was no significant difference between men and women in “student-patient” or in “self-colleagues” factor (Figure 1, Panel 1A). There was a significantly higher score among women as compared to men for item 9: “The program helped me process the emotional impacts of my experiences in the medical ward” (4.93 ± 1.81 vs. 4.11 ± 1.88, p = 0.03).

### Students’ ethnicity and the evaluation of the program’s effects

Among Arab and Druze students there was a significantly higher scores for factor 1 (“student-patient”) (5.66 ± 0.98 vs 4.81 ± 1.52, p = 0.043), while there was no significant difference for factor 2 (“self and colleagues”), (p = 0.361), (Figure 1, Panel 1B). Scores on items 1 and 5 that are included in factor 1 were significantly higher among Arab and Druze students as compared with Jewish students [Item 1: “The program increased my awareness of how patients feel” (5.87 ± 0.99 vs. 5.27 ± 1.63, p = 0.001), item 5: “The program motivated me to increase my efforts towards patients” communication” (4.80 ± 1.82 vs. 3.70 ± 1.76, p = 0.03)], while in two additional items included in factor 1 (items 4 and the negatively worded item 2) there was a non- significant trend towards a higher evaluations among Arab and Druze students (both p values <0.07).

### Students’ attitude towards poetry and the evaluation of the program’s effects

Average ± SD score on “Generally, I enjoy reading poetry” was 4.70 ± 1.99, and median was 5. In 51 participants the score was >5, and in 61 the score was ≤5. Students’ attitudes towards poetry-reading was related to gender [significantly higher proportion of women with score >5 as compared with men (p = 0.014)]. There were no association between attitude towards poetry-reading and students’ ethnicity, or timing of the program (p values >0.71).

Factor 1 (items 1-5) score was not significantly associated with students’ attitude towards poetry. However the average score for factor 2 (“Self and colleagues”, items 6-9) was significantly higher among students with more positive attitude towards poetry (4.96 ± 1.38 vs 4.11 ± 1.61, p = 0.004), (Figure 1, Panel 1C). Three of the four items included in factor 2 were related with the attitude towards poetry. These include: Item 6: “The program provoked conversations among students in topics that are related to the clinical experiences” (5.02 ± 1.75 vs 4.07 ± 2.04, p = 0.008), and item 9: “The program helped me process the emotional impact of my experiences in the medical ward” (4.92 ± 1.87 vs 3.97 ± 1.69, p = 0.006), respectively. In the negatively worded item 7: “The program did not affect students’ communication regarding clinical experiences”, the opposite was found (2.61 ± 1.65 vs 3.77 ± 2.16, p = 0.002).

### Timing of the program and the evaluation of the program’s effects

There was no difference in factor 1 (“student-patient”) between students who participated in the program during their first and second 4^th^ year clerkship. However factor 2 (“self and colleagues”) was higher among students who participated in the program during their second 4^th^ year clerkship, with borderline statistical significance (4.78 ± 1.46 vs 4.20 ± 1.61, p = 0.047) (Figure 1, Panel 1D).

### Mode of students’ participation

Students’ scores on “Participation should be obligatory” were significantly higher as compared to “The program should be offered to students as an elective course” (4.66 ± 1.97 and 3.05 ± 1.83, respectively, p < 0.0001, paired Student t test), suggesting that students favored obligatory rather than optional participation in the program.

There was no statistically significant gender or ethnic difference in items regarding students participation in the program [“Participation should be obligatory”: males: 4.54 ± 1.99, females: 4.83 ± 1.99, p = 0.49; Arabs and Druze 4.09 ± 2.37, Jewish 4.79 ± 1.87, p = 0.213. “The program should be offered to students as an elective course”: males: 3.25 ± 1.65, females: 2.64 ± 1.91, p = 0.096; Arabs and Druze 2.47 ± 1.25, Jewish 3.00 ± 1.82, p = 0.283]. Scores on items regarding mode of participation in the program were not related with students’ attitude towards poetry-reading (All p values >0.25).

There was a positive correlation between students’ scores on “Participation should be obligatory” and the average scores of factor 1 and 2 [Pearson correlations: 0.21 (p = 0.034), 0.238 (p = 0.013), respectively], and a negative correlation between Students’ scores on “The program should be offered to students as an elective course” and the average scores of factor 1 and 2 [Pearson correlations: -0.36 (p < 0.0001), -0.34 (p < 0.0001), respectively].

## Discussion

We examined students’ characteristics associated with the evaluation of various aims of a clinically-integrated art-based intervention, in an unselected student population. Our main findings are that of the two effects of the intervention that were identified, students’ evaluation of the effect of the intervention on their relationship with patients (“student-patient”) was independent of their own attitude towards poetry-reading. The evaluation of this factor was higher among Arab and Druze as compared to Jewish students.

In contrast to the evaluation the effect on relationship with patients (“student-patient”), students’ evaluation of the effect of the program on self-reflection and communication with colleagues (“self and colleagues”) was associated with the individual attitude towards poetry-reading and with the timing of the program late during the 4^th^ year studies. Students’ gender was not associated with any of the effects identified. Among the entire student group, we observed a significantly higher score for “student-patient” factor, as compared to the “self and colleagues” factor.

Although recognized as an essential part of medical education [15–17], medical students’ empathy was found by some authors [17–19], but not by others [20], to decrease during medical studies. Art-based interventions have been suggested as a possible means by which students’ empathy can be enhanced [1, 2]. However, whether students’ individual attitudes towards the arts are related with their evaluation of relationship with patients, or with other effects of a required art-based intervention, has not been examined.

Our findings, that students’ individual attitudes towards poetry-reading were not related to their evaluation of the impact of COPE on the “student-patient” effect, while it was related with the “self and colleagues” effect, may have potential implications on curriculum planning, specifically regarding the format of students’ participation in art-based programs. While medical humanities have often been included in the medical curriculum in the form of elective, rather than as required courses [3, 5–9], data on required art-based interventions are limited. In a study evaluating an elective medical humanities program [9], students who were assigned to the program because their first choice elective was not available, confirmed that the course had been extremely valuable to them [9]. In our study, students preferred a format of obligatory rather than elective sessions, and these preferences were not related to their attitude towards poetry-reading.

Overall, our findings suggest that a format of integrated, obligatory poetry-based intervention may be used for enhancing “student-patient” effects in heterogeneous student populations. Nevertheless, our study suggests that students’ evaluation of aims of art-based interventions regarding “self and colleagues” effects are affected by their attitude towards poetry more than “student-patient” aims. Thus, a combination of approaches, such as elective and non-elective sessions and courses, may be required to address various aims of art-based interventions in medical education [1–3].

We studied an ethnically heterogeneous population, including Jewish and Arab students. Since the effects of art-based curricula are likely to be culture - dependent, ethnic variability could effect the evaluation of poetry-based programs. Ethnic differences in the evaluation of medical humanities programs have not been previously reported. We found higher evaluations of the program’s impact on “student-patient” effect among Arab and Druze students, as compared with Jewish students. This may be related to cultural differences in the perception of the “student-patient” factor in medical professionalism or to differences in the attitude towards poetry. However, in our study the attitude towards poetry-reading was not associated with student ethnicity. The findings of this study suggest that in the population studied the evaluation of poetry-based curricula was not limited by differences between students’ ethnicity and the ethnicity of the poems’ authors. Nevertheless, the ethnic difference observed in this study increased our awareness of the potential implications of ethnic diversity on the programs’ outcomes, and since we have previously used Hebrew-written poetry in this program we increased the use of translated poetry and literature.

We observed a higher evaluation of “self and colleagues” factor among students who participated in the program on their second 4^th^ year clerkship. However, no timing difference was found for the “patient-student” factor. Medical students’ empathy towards patients was shown to decline following their initial extensive clinical experiences in medical school [19]. This is why we integrated COPE into the fourth year of medical school which is the first “clinical” year of studies. The lack of difference in the evaluation of the “student-patient” factor during the fourth year may be because the time difference between the first and the second clerkship may not have been large enough to allow the detection of such potential effect. The higher evaluation of the “self and colleagues” factor among students who participated in the program during their second 4^th^ year clerkship may be related to an increasing role of student group during this academic year, possibly due to growing trust and collegiality within group during 4^th^ year studies. However, specific study is required to address this question.

There are several potential explanation the higher evaluation of “student-patient” factor as compared to the “self and colleagues” factor. First, the poems used in this program were related to illness, death, patients and caregivers, and cultural issues, but not with interactions amongst students. In addition, it is possible that “student-patient” aims (empathy and communication with patients) were perceived by students as related to the core of medical professionalism more than aims related to “self and colleagues” (self-reflection and communication), resulting in higher evaluation of the former as compared to the latter.

Our study has several limitations. We report on students’ evaluation of a medical humanities program, and the effect on student performance was not monitored. However, previous studies on students’ evaluation of art-based interventions were small qualitative studies and none has included an evaluation of students’ performance [4]. In addition, no previous study has examined the effect of students’ characteristics, including ethnicity and gender, in an unselected student population on the evaluation of such interventions.

We used questionnaires that were constructed to represent main domains according to previously suggested impacts of medical humanities, and factor analysis was performed as evidence of internal structure [11, 12]. Our finding, that items regarding “student-patient” effect (relationship with patients) clustered separately from other effects (such as self-reflection and communication with colleagues), is in accordance with the previously suggested taxonomy of the effects of medical humanities [2].

We analyzed the data of the pilot together with the data of the following years. The CFA in 112 students yielded similar results to the analysis in 67 students. In addition, the aims and methods of the program did not change substantially throughout the study, and the same evaluation questionnaire was used during the three study years (2008 -10).

Another consideration is our decision to use poetry in COPE. Poetry was suggested to require competencies that are analogues to those required in medicine, such as linguistic sensitivity, cultural awareness and the ability to interpret human situations [21–24]. The poems used in COPE were short and immediately accessible. Thus, students were not required to prepare for the activity in advance. Since other forms of humanities were not applied in COPE, our conclusions are currently limited to poetry-based interventions, and to poems that are related to clinical experiences.

Integration of medical humanities programs within the clinical clerkship has several potential advantages. These include the role modelling of clinical instructor [25] and the clinical context of the program [3]. COPE instructors are internal medicine board-certified junior faculty. Students meet these physicians during the clerkship on other occasions then COPE, such as during ward rounds and patients’ consultations. Thus, the "hidden curriculum" of COPE clinical orientation could demonstrate to students the continuum of biomedical and interpersonal components of physicians’ professionalism [17].

## Conclusions

We identified two effect factors of COPE: “student- patient” and “self and colleagues”. Arab and Druze students gave higher scores to “student-patient” factor as compared to Jewish students. Students’ own attitude towards poetry-reading was not related with their evaluation of the program’s impact on “student-patient” effect, but was related with the evaluation of the “self and colleagues” effect. In addition, higher evaluation of the “self and colleagues” factor was observed among students who participated in the program late as compared to early during the 4^th^ year studies. Ethnic differences, variability in individual’s attitude towards the arts and timing of the program should be considered when planning art-based educational interventions. COPE may be included in the medical curriculum in order to enhance “student-patient” aims as an obligatory course, possibly in combination with elective courses/activities. Further study is required to determine the effect of art-based interventions on actual empathy of medical students in patient encounters.
